# Posterior Scleritis in a Pediatric Patient: Importance of Pain Recognition and Early Ultrasound Diagnosis

**DOI:** 10.7759/cureus.63369

**Published:** 2024-06-28

**Authors:** Ahmed B Alsatrawi, Amani Alzayani

**Affiliations:** 1 Ophthalmology, Salmaniya Medical Complex, Manama, BHR

**Keywords:** uveitis, pediatric, optic neuritis, b-scan ultrasonography, headache, posterior scleritis

## Abstract

Scleritis is a rare and potentially sight-threatening inflammatory disorder affecting the sclera. While anterior scleritis is the more commonly encountered subtype, posterior scleritis represents a larger portion of cases in the pediatric population. Diagnosing posterior scleritis can be challenging due to the overlap with other more common ocular conditions, particularly in the pediatric age group.

In this case report, we present a unique case of posterior scleritis in a 12-year-old girl. The patient initially presented with severe right-sided headache and eye pain, which was initially misdiagnosed as atypical optic neuritis. However, subsequent magnetic resonance imaging and ocular ultrasound confirmed the diagnosis of posterior scleritis.

This case highlights the significance of recognizing pain as a key symptom and the value of early ultrasound evaluation in diagnosing posterior scleritis in the pediatric population.

## Introduction

Scleritis is a rare and potentially sight-threatening inflammatory disorder affecting the sclera, commonly characterized by pain and engorgement of deep scleral vessels [1]. It can involve either the anterior or posterior sclera, with the anterior subtype being by far the more commonly encountered type and the posterior the less frequent [2]. However, it is noteworthy to know that this ratio changes in the pediatric population where posterior involvement appears to have a higher rate of occurrence reaching up to 50% of all cases of pediatric scleritis in some reports [2,3].

Diagnosing posterior scleritis can be challenging sometimes due to the overlap with other more common ocular conditions and this becomes more difficult in the pediatric age group [3]. In this case report, we present a unique case of posterior scleritis in a pediatric patient, emphasizing the significance of identifying pain as a symptom and the value of early ultrasound in the diagnosis of posterior scleritis to avoid unnecessary investigation and faster disease control.

## Case presentation

A 12-year-old healthy young girl presented on 28/1/2024 to the main accident and emergency unit with a history of severe right-side headache that awakened her from sleep for one week, associated with nausea, vomiting, and right eye tearing. A basic clinical examination, blood workup, and brain computed tomography (CT) were normal, and the patient was discharged on oral analgesics.

One week later on 5/2/2024, the patient came back to the main accident and emergency with the same complaint but the headache and pain being more toward the right eye. Repeated basic clinical examination, blood workup, and brain CT were all normal.

**Figure 1:**
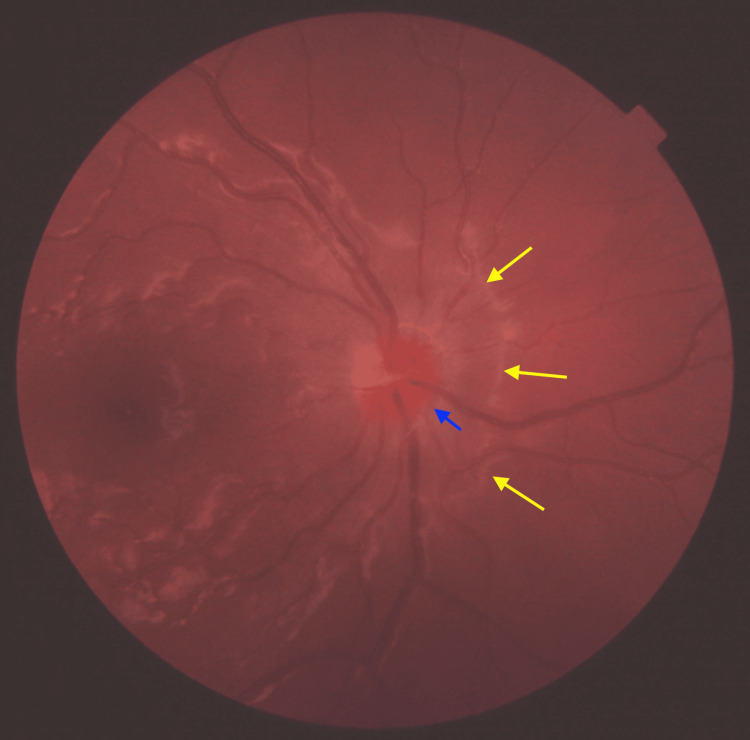
Color fundus photo of the right optic disc (pupil not dilated/limited brightness). Hyperemic optic disc (blue arrow) with elevation reaching the peripapillary area (yellow arrows).

The patient on this visit was referred to the ophthalmologist on call. The ophthalmic examination concluded with a provisional diagnosis of right eye atypical optic neuritis, being supported by the increase in severity of pain with eye movement, hyperemic disc swelling (Figure 1), and inconclusive weak relative afferent pupillary defect.

**Figure 2:**
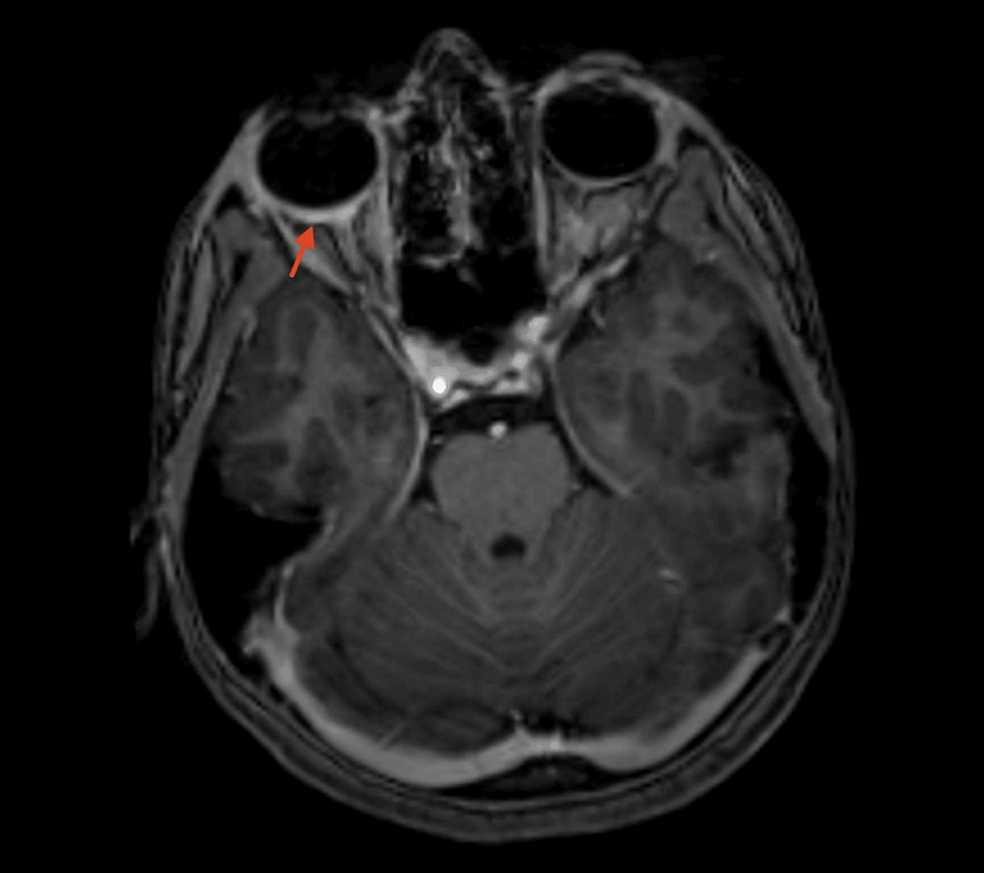
MRI of the brain and orbit (axial T1 post-contrast image). Thickening and enhancement of the posterior sclera of the right eye (red arrow).

**Figure 3:**
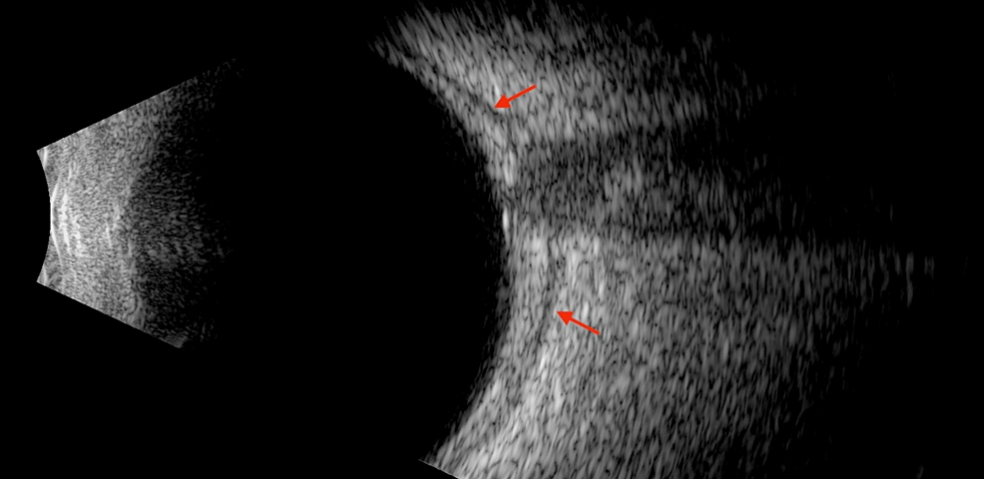
Ophthalmic ultrasound (B-scan) of the right eye. Positive T-sign (red arrows).

The patient was admitted for intravenous (IV) methylprednisolone 120 mg every six hours for three days and magnetic resonance imaging (MRI) of the brain and orbit. Imaging was done on the second day of admission and showed findings highly suggestive of right-eye posterior scleritis associated with retrobulbar and perineural changes suggestive of inflammation for clinical correlation (Figure 2). An ocular ultrasound (B-scan) done later in the day showed the classical T-sign and confirmed the diagnosis of posterior scleritis (Figure 3).

She was diagnosed with posterior scleritis and showed significant improvement in her symptoms after three days of IV methylprednisolone. She was discharged on 40 mg of oral prednisolone (1 mg/kg) with a plan of slow taper over the course of six months. She is currently in her 4th month on 6 mg prednisolone, is symptom-free, and all her examinations and imaging are back to normal.

## Discussion

Scleritis is relatively rare, with an estimated incidence ranging from around three to five cases per 100,000 individuals per year [4-6]. It predominantly affects adults, but infrequent cases in the pediatric population are seen too.

The Watson system classifies scleritis according to the anatomical location into five categories: diffuse anterior, nodular anterior, necrotizing anterior without inflammation, necrotizing anterior with inflammation, and posterior scleritis [7].

Diffuse and nodular anterior types of scleritis are by far the most common [8,9]. Posterior scleritis refers to inflammation of the sclera posterior to the ora serrata and is estimated to account for only 2% to 12% of all cases [1]. Incidence of posterior scleritis in children is even rarer than in the adult population. However, it represents a larger portion of the overall incidence of scleritis in this age group [1,3,10].

Prompt diagnosis and management of posterior scleritis is essential to prevent potential vision-threatening complications and avoid long-term irreversible vision loss.

Pain may be less prominent in posterior scleritis than in anterior scleritis, leading to potential diagnostic challenges, particularly in pediatric patients who may have difficulty expressing their symptoms [1,3,10]. However, it is almost always missed for other ocular inflammations associated with pain, e.g., anterior scleritis, episcleritis, iritis, optic neuritis, and orbital inflammatory disorders [1]. Majumder et al. reported pain as being the most common presenting complaint in pediatric scleritis (90%), followed by redness in 60% of patients [3]. Painless posterior scleritis has been reported previously [11], but despite that, pain remains the hallmark presenting symptom in scleritis and its recognition is crucial for early diagnosis and appropriate treatment.

Ophthalmic ultrasound (B-scan) plays a vital role in confirming the diagnosis and evaluating the extent of inflammation in posterior scleritis. It allows the visualization of the thickened scleral and the retrobulbar edema and fluid collection, which is the pathognomonic T-sign in posterior scleritis [10,12,13]. Cheung and Chee reported in one of the largest case series of pediatric patients with posterior scleritis, where all eyes (100%) demonstrated positive T-sign on B-scan ultrasound [1]. It is a non-invasive and cost-effective modality that can be particularly beneficial in pediatric patients, aiding in early accurate diagnosis.

## Conclusions

This case report highlights the presentation of posterior scleritis in a pediatric patient and underscores the importance of early recognition of pain as a symptom in posterior scleritis, especially in cases where the clinical examination and the provisional diagnosis cannot explain the extent and severity of pain. Additionally, it emphasizes the importance of the early utility of ophthalmic ultrasound as a valuable diagnostic tool in posterior scleritis, especially in pediatric patients. It avoids unnecessary investigations that can be costly and harmful to the child and provides fast clues of diagnosis to allow early treatment for symptomatic relief and complication prevention. By sharing this case, we aim to enhance awareness of posterior scleritis in the pediatric population, emphasize the significance of pain in its diagnosis, and promote the utilization of ophthalmic ultrasound as an important diagnostic modality in posterior scleritis.
